# Submit a Topic Page to *PLOS Computational Biology* and Wikipedia

**DOI:** 10.1371/journal.pcbi.1006137

**Published:** 2018-05-31

**Authors:** Daniel Mietchen, Shoshana Wodak, Szymon Wasik, Natalia Szostak, Christophe Dessimoz

**Affiliations:** 1 Data Science Institute, University of Virginia, Charlottesville, Virginia, United States of America; 2 Vlaams Instituut voor Biotechnologie-Vrije Universiteit Brussel Centre for Structural Biology, Brussels, Belgium; 3 Institute of Computing Science, Poznan University of Technology, Poznan, Poland; 4 European Centre for Bioinformatics and Genomics, Poznan University of Technology, Poznan, Poland; 5 Institute of Bioorganic Chemistry, Polish Academy of Sciences, Poznan, Poland; 6 University College London, London, United Kingdom; 7 University of Lausanne, Lausanne, Switzerland; 8 Swiss Institute of Bioinformatics, Lausanne, Switzerland

Back in March 2012, *PLOS Computational Biology* launched its ‘Topic Pages’ project as a way to help fill important gaps in Wikipedia’s coverage of computational biology content and to credit authors for their contributions. Topic Pages are written in the style of a Wikipedia article and are openly and publicly peer reviewed on the PLOS Wiki before being published in our PLOS journals, with a second, editable version posted to Wikipedia.

Six years on, *PLOS Computational Biology* has published 11 Topic Pages covering a good range of subjects, from the Hypercycle to Approximate Bayesian Computation. The published articles have been widely viewed on Wikipedia as well as in the journal and well received by the community.

We are welcoming submissions for further *PLOS Computational Biology* Topic Pages. We are looking for topics in computational biology that are of interest to our readership, the broader scientific community, and the public at large and that are not yet covered or insufficiently covered (i.e., exist as a ‘stub’) in Wikipedia. Last year, *PLOS Genetics* joined the Topic Pages initiative, as detailed in this blog post.

We are also exploring how the Topic Pages approach could be extended to include Wikidata, the community-curated database connecting concepts covered in any Wikipedia article with the Semantic Web [1]. For instance, data from more and more research-related databases are being integrated with Wikidata or its semantic core, Wikibase. This creates the need to formalize data models: How should concepts like a disease outbreak, a cell-cycle checkpoint, a sequencer, biomineralization, or a functional magnetic resonance imaging (fMRI) data set be modelled in Wikidata or Wikibase? Conversely, what workflows allow us to collect information about such concepts in Wikidata, to interlink it with related information, to validate it, and to keep it up to date? Or, how can the data from Wikidata be explored or put to use in other contexts relevant to computational biology? We are working on establishing the editorial workflows to handle such Wikidata-focused Topic Pages and would welcome submissions to test these waters. For some inspiration, we suggest taking a look at Wikidata-based tools for browsing microbial genomes [2], scholarly publications [3], or software and file formats [4].

The Author Guidelines for Wikipedia-focused Topic Pages are available here. If you’ve noticed a gap in Wikipedia’s coverage of particular computational biology topics, we want to hear from you! Please send ideas for Topic Pages to ploscompbiol@plos.org.

Read on to find out more about how Topic Pages work from authors Szymon Wasik, Natalia Szostak, and Christophe Dessimoz, who describe their experiences of the editorial process and the lives of their articles post-publication.

## Szymon Wasik and Natalia Szostak

During work on one of our recent projects [5], myself and my colleagues searched for sources describing a key concept that our work touched upon—the hypercycle. We found out that there is a vast amount of literature on this subject written by excellent researchers, but despite this, and despite the undeniable importance of the hypercycle theory for the computational and mathematical approaches related to the origin of life, a comprehensive summary of current knowledge on the topic was missing. Moreover, when we turned our attention to Wikipedia, we realized that the ‘Hypercycle’ article was insubstantial: a short note that clearly needed improvement. At the same time, we discovered that *PLOS Computational Biology* has a ‘Topic Pages’ project that encourages authors to write Wikipedia articles on computational biology-related topics. As we are Wikipedia enthusiasts and like the spirit of sharing knowledge, we thought that the effort we had put into researching literature on the hypercycle during our project could be well used to prepare a ‘Hypercycle’ Topic Page [6].

**Fig 1:**
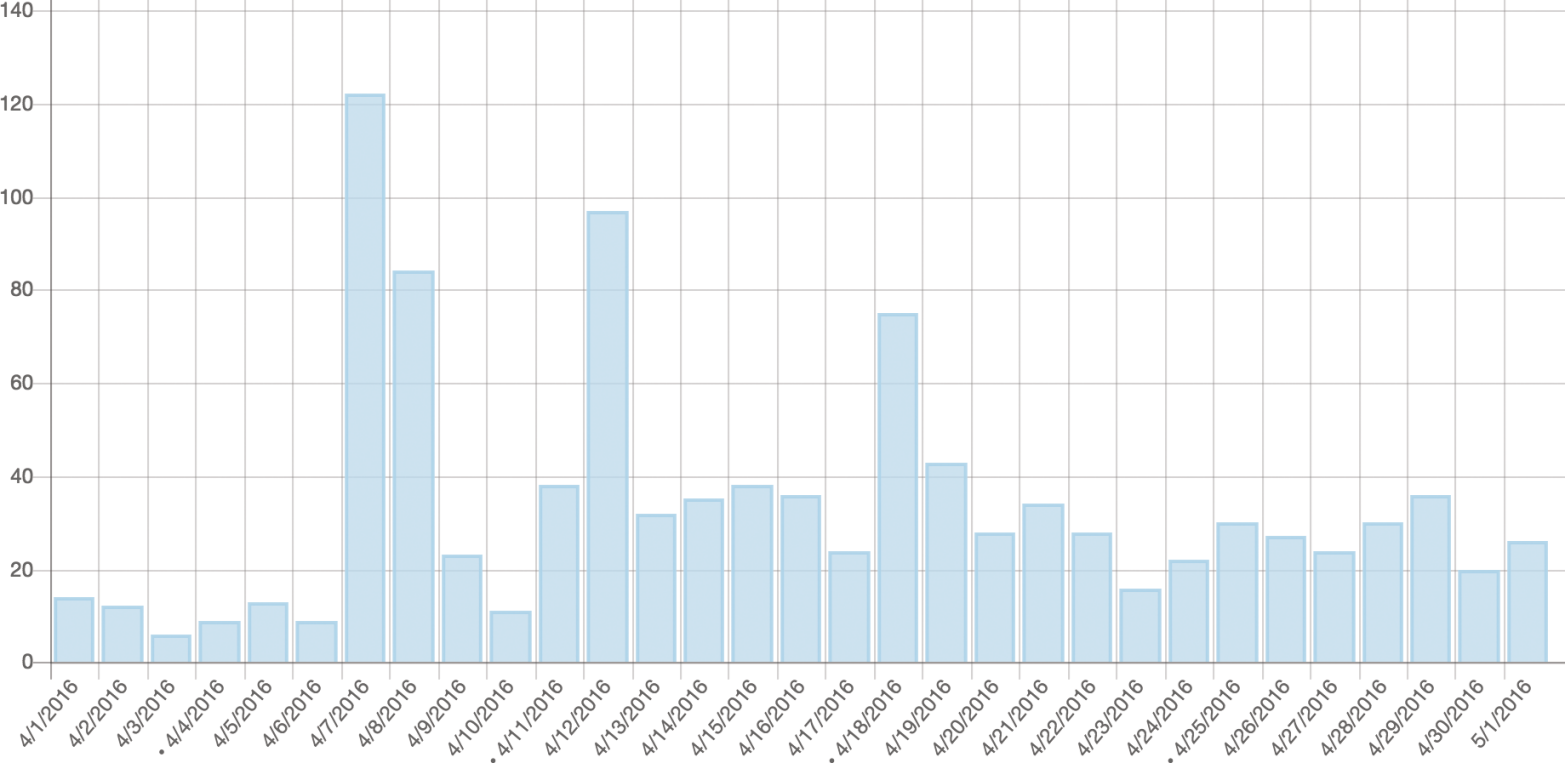
Increase in the popularity of Hypercycle article after being published by *PLOS Computational Biology* (number of visits per day).

Now, if you think that adding a Hypercycle article to Wikipedia is a piece of cake, you’re mistaken! Given the rich source materials, some inconsistencies, and misunderstandings, we spent many hours reading and unifying the knowledge. Adding the sophisticated edition criteria set by Wikipedia, it was probably the hardest article that we have ever written. Luckily, during the whole process, we received a lot of help from *PLOS Computational Biology* Topic Pages editor Daniel Mietchen in adapting the text to Wikipedia standards. When the article was ready for review, it went through a rigorous evaluation process by excellent researchers. We very much enjoyed the open peer-review and revision process. Thanks to the reviewers, the peer-review process was more like a discussion than a set of criticisms that is often prepared by referees. After being accepted by *PLOS Computational Biology*, the editors seamlessly transferred the article to Wikipedia. All of these efforts paid off, as after publishing the Hypercycle Wikipedia article in April 2017, the viewership of its content doubled (see Fig 1).

**Fig 2:**
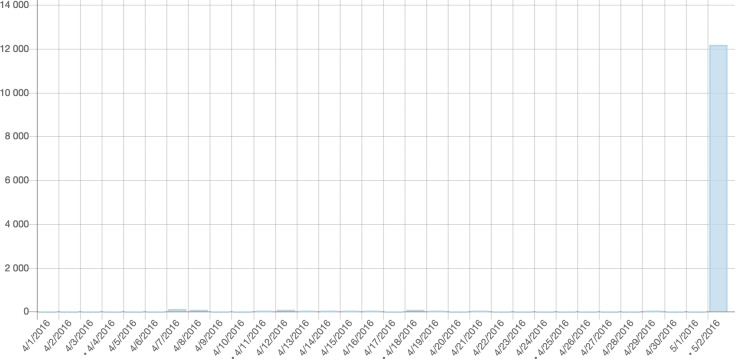
Number of visits to Hypercycle article after being promoted.

However, it was not the end of our work on the article. We wanted to make use of the chance to promote the page using the ‘Did you know…‘ section on the Wikipedia homepage, which highlighted important new or significantly improved articles for 24 hours (in 2016 it was 12 hours). To be accepted for this section, we had to pass a strict review process by experienced Wikipedia editors. This process helped us to increase the quality of the article even more, primarily by adjusting the style and references to Wikipedia standards. When the page was finally promoted on 2 May 2016, it was visited by over 12,000 people in a single day, becoming the third most popular ‘Did you know …’ article in May (see Fig 2).

Thanks to the ‘Topic Pages’ initiative, the hypercycle theory is now more accessible not only for advanced readers, but also for ordinary people who seek knowledge on the computational aspects of the origins of life. The Wikipedia Hypercycle page and its rich reference base is now a good starting point for further research. And as for us, we feel delighted that we could be a part of a project that brings knowledge to a broader audience.

## Christophe Dessimoz

Our lab was involved in publishing two Topic Pages: one on Approximate Bayesian Computations published in 2013 [7] and one on Inferring Horizontal Gene Transfer in 2015 [8]. As an encyclopaedia, Wikipedia is a form of tertiary literature, and thus Topic Page articles should be relatively general—mainly building on review articles—and be written as neutrally as possible. At the same time, Topic Pages are also published in *PLOS Computational* Biology as peer-reviewed articles, so they should also make a meaningful contribution to the scientific literature.

Now that a few years have passed, we can attempt to evaluate the impact of our Topic Pages on Wikipedia and on the scientific literature. For Wikipedia, the main impact is that our Topic Pages provided fully fledged articles where there had previously been very little coverage (Approximate Bayesian Computation) or even no coverage at all (Inferring Horizontal Gene Transfer). Access statistics [9] indicate that the pages are regularly consulted, with 27,400 and 4,400 page views in 2017. As a point of comparison, the ‘Computational Biology’ Wikipedia page had 114,000 views during that year.

Famously, Wikipedia articles can be updated by any reader. Since publication, our two articles were updated 126 and 34 times. Overall, these changes were minor—mainly spelling and formatting improvements. In my experience, this is less than what a typical peer review would require! I don't think this is because our articles are timeless, but rather because for our topics, the domain experts are scientists, and most scientists are still not used to contributing to Wikipedia.

For the research community, the impact of our two Topic Pages can be gauged, admittedly crudely, by bibliometrics. According to Google Scholar, the number of citations are 204 and 57. These solid figures suggest that the articles have been useful.

Thus, our two Topic Pages have fulfilled their purpose both for Wikipedia and for the scientific community and as a result proved very gratifying for us.

The editorial process itself was great. Our editor, Daniel Mietchen, helped tremendously to bring the article up to Wikipedia's standards. And in true Wikipedian spirit, he made many edits on the manuscript directly. Likewise, the open, wiki-based peer-review process felt more constructive and straightforward than the standard model, in which communication between reviewers and authors is channelled through the editor.

In summary, I am a fan of the Topic Page format. If your domain of expertise is poorly covered on Wikipedia, consider contributing one! Particularly if you are a student or an early career researcher, publishing a Topic Page can be a great opportunity to publish a high-impact paper and to develop important writing skills by producing an accessible, jargon-free Wikipedia article. For a more detailed account of our experience as Topic Page authors and additional thoughts on the (complicated) relationship between academia and Wikipedia, you can read the blog post that I wrote a few years ago [10].
